# From sunscreens to medicines: Can a dissipation hypothesis explain the beneficial aspects of many plant compounds?

**DOI:** 10.1002/ptr.6654

**Published:** 2020-03-12

**Authors:** Alistair V.W. Nunn, Geoffrey W. Guy, Stanley W. Botchway, Jimmy D. Bell

**Affiliations:** ^1^ Research Centre for Optimal Health, Department of Life Sciences University of Westminster London UK; ^2^ GW pharmaceuticals Salisbury Wiltshire UK; ^3^ STFC, UKRI & Department of Biological and Medical Sciences Oxford Brookes University Oxford UK

**Keywords:** ageing, cannabidiol, curcumin, hormesis, inflammation, mitochondria, origins of life, quantum, quercetin, resveratrol, salicylic acid, sunscreens, thermodynamics, UV

## Abstract

Medicine has utilised plant‐based treatments for millennia, but precisely how they work is unclear. One approach is to use a thermodynamic viewpoint that life arose by dissipating geothermal and/or solar potential. Hence, the ability to dissipate energy to maintain homeostasis is a fundamental principle in all life, which can be viewed as an accretion system where layers of complexity have built upon core abiotic molecules. Many of these compounds are chromophoric and are now involved in multiple pathways. Plants have further evolved a plethora of chromophoric compounds that can not only act as sunscreens and redox modifiers, but also have now become integrated into a generalised stress adaptive system. This could be an extension of the dissipative process. In animals, many of these compounds are hormetic, modulating mitochondria and calcium signalling. They can also display anti‐pathogen effects. They could therefore modulate bioenergetics across all life due to the conserved electron transport chain and proton gradient. In this review paper, we focus on well‐described medicinal compounds, such as salicylic acid and cannabidiol and suggest, at least in animals, their activity reflects their evolved function in plants in relation to stress adaptation, which itself evolved to maintain dissipative homeostasis.

## 1 INTRODUCTION

To date, there is still no real consensus on why many plant products exert their medicinal benefits in animals, but certainly compounds such as salicylic acid, resveratrol, curcumin, the green tea catechins, and the phytocannabinoids, tetrahydrocannabinol (THC) and cannabidiol (CBD), appear to be anti‐inflammatory and anti‐proliferative. Although many do have established intracellular receptors and targets (Duthie & Wood, 2011; Itokawa, Shi, Akiyama, Morris‐Natschke, & Lee, 2008; Patra, Rizzi, Silva, Rugina, & Bettuzzi, 2008; Pertwee, 2014; Pervaiz & Holme, 2009), the sheer number has made defining their mode of action difficult. For instance, the number of targets and pathways that resveratrol interacts with is bewildering, as multiple groups have found that it modulates inflammation, redox, cell cycle, death and survival, kinases, mitochondrial function, autophagy and affects multiple receptors, transcription factors and ion channels; the outcome is often biphasic, depending on dose (Pervaiz & Holme, 2009). Equally, more than 65 discrete molecular targets have been reported in the literature for CBD (Ibeas Bih et al., 2015). The number of targets identified and predicted for salicylic acid also continues to grow (Alfonso, Ai, Spitale, & Bhat, 2014). Hence, the possibility that a single ‘druggable’ target could explain how medicinal plant compounds work in animals has become less plausible, but does suggest they could be ‘multi‐target’, and the diverse number of systems they seem to be interacting with being explained by evolution building on their basic properties and the shared biochemistry between plants and animals.

A key clue, we believe, is embraced by the concept of ‘xenohormesis’: in effect, because most of these compounds are related to stress adaptation in the plant, animals have adopted them as they provide environmental signals that can help them survive more difficult times (Hooper, Hooper, Tytell, & Vigh, 2010; Lamming, Wood, & Sinclair, 2004). Although the activation of the xenobiotic system can explain many of the beneficial effects (Mattson, 2008; Zhang, Pi, Woods, & Andersen, 2009), it could also be related to ‘mitohormesis’ as many of these compounds stimulate mitochondrial reactive oxygen species (ROS) as an adaptive signal (Tapia, 2006). In fact, many polyphenols do seem to modulate mitochondrial function (Gorlach, Fichna, & Lewandowska, 2015) and can initiate mitophagy (Tan & Wong, 2017). Critically, many plant extracts show biphasic effects in animal models, that is, at low doses, they seem to be pro‐inflammatory, but at higher doses, they become anti‐inflammatory, which is commensurate in some cases with an increase in toxicity (Schink et al., 2018) and is thus strongly suggestive of hormesis (Calabrese, Agathokleous, Kapoor, Kozumbo, & Rattan, 2019). Furthermore, data also indicate many of these compounds are also are anti‐bacterial (Daglia, 2012), which might hint that their ability to also modulate mitochondria may share a common mechanism. The key point is that central components of the electron transport chain (ETC), and the proton gradient, were among the first systems to evolve after life began and are retained in all life and have become more complex, but only by accretion of extra components.

The origins of these abilities seem to have arisen as plants started to colonise the land and thus had the need to resist much higher levels of UV and the generation of free radicals by photon absorption. To do this, they evolved a plethora of chromophoric compounds containing conjugated double‐bond systems that cannot only absorb light efficiently, potentially acting as sunscreens, but also appearing to act as antioxidants—thus modulating ROS‐based signalling (Brunetti, Fini, Sebastiani, Gori, & Tattini, 2018). This may be supported by observation that the production of most medicinally useful plant compounds is generally increased by exposure of the plant to UV, including *Cannabis sativa (*Zhang & Bjorn, 2009).

However, like nearly all life, plants are largely dependent on the generation of a proton gradient to generate ATP, which is itself driven by electrons flowing through a highly conserved ETC: the main difference between photosynthesis and respiration is the source of electrons. Critically, as over‐reduction of the ETC can create damaging free radicals, as can collapse of the proton gradient, this system evolved complex mechanisms to control both proton and electron leak, and thus, adaptive signalling; for instance, mitochondria may need to work at an intermediate redox state (Aon, Cortassa, & O'Rourke, 2010; Cortassa, O'Rourke, & Aon, 2014). This might explain why mitochondria and chloroplasts retain genes that are part of a localised redox regulatory system (Allen, 2015). This seems to put mitochondria centre stage in determining ageing and pathology, and thus, lifespan (Lane, 2005).

In this paper, we outline the idea that the base property of many secondary plant metabolites is that they can both directly, and indirectly, modulate mitochondrial function because they represent an ancient structural principle, which was possibly driven by presence of UV and a proton gradient. This was the creation, and then selection, of compounds containing conjugated double bond systems that have the dual ability to both absorb light efficiently, and potentially act as sunscreens, but also modulate redox, and where necessary, generate an adaptive signal. These properties have been amplified and adopted, in effect, accreted, from abiotic geothermal/light driven chemistry via addition of more complex protein/lipid‐based systems to ensure homeostasis in an ever‐changing environment. Evolution has, through its ‘tinkering’ (Jacob, 1977), found many new uses for them to enhance resistance to stress. In short, if life can be viewed as a ‘dissipative’ structure, then these compounds can fine tune this process in response to environmental stress.

In terms of medicine, a key component of ageing and many diseases is chronic inflammation (Furman et al., 2019), which can be viewed as the decrease in efficacy of a feedback system that controls the flow of electrons. Inflammation, it could be said, simply represents an evolved system that is triggered when electron flow is altered, either by damage, infection or other severe environmental stress, to correct the problem. It therefore represents a hormetic system, as the stress initiates an adaptive programme that should resolve it, if it does not, then the system eventually fails and is thus subject to natural selection. Thus, controlling it is a priority. Hence, the modulation of inflammation by many plant compounds could be explained by their evolved function in stress protection: at low doses they would amplify a ROS‐based signal (in effect, an electron leak), which in some systems would appear to be inflammatory. However, with time, and/or higher doses, this would shift towards being anti‐inflammatory to limit the response to prevent a vicious positive feedback cycle. This would be associated with an increased capacity to flow electrons safely, for instance, by enhancing mitochondrial capacity, and thus the ability to dissipate energy or excess electrons. This paradigm would be reflected in their ability to inhibit proliferation of cancer cells and pathogens, which are problems common to both plants and animals. This could be explained by their basic physical properties to ‘dissipate’ energy, and by subsequent evolution of proteins that enhanced these principles, and by life in general being a dissipative structure.

## 2 ORIGINS OF LIFE THEORIES AND EMERGING CONCEPTS

In order to understand how plant secondary metabolites are functioning as medicines, it may be helpful to review some theories on the origins of life as extant biochemistry reflects both pre‐life geochemistry and photochemistry. This of course is generally a speculative subject, as there are still several competing theories, such as the genetics first idea (replication first in warm ponds, for instance), metabolism first (thermal vents), as well as vesicle first (lipids), with many of the chemicals necessary being delivered from inter‐ and circumstellar environments (Jheeta, 2016). Most of these models rely on the existence of a ‘privileged function’, such as replication for the ribonucleic acid (RNA) world, in metabolism first models, it is autocatalytic networks in an energy gradient, such as a hydrothermal vent, and in membrane worlds, compartmentalisation. Another way of looking at this is the universal gene set and the molecular toolbox—that is, which components are common to all life suggesting its most ancient requirements, such as 53 universal genes involved in translation, 28 amino acids, coenzyme A, nicotinamide adenine dinucleotide (NAD) and certain ribonucleotides (Lanier & Williams, 2017). Plus, all of these would have embraced the control of ions such as calcium, which itself seems to have been a key player in evolution (Kazmierczak, Kempe, & Kremer, 2013; Plattner & Verkhratsky, 2015).

Two major theories involve life starting in an alkaline thermal vent and some variations of a UV‐driven process, with the possibility of some amalgam of these. What is clear is that both a proton gradient, and chemistry based on UV‐absorbing compounds, is critical today. There are many theories on the origins of life, and it is beyond the scope of this article to review them all, but the reader is directed to books such as that by Smith and Morowtiz (Morowitz & Smith, 2016) or Egel, Lankenau and Mulkidjanian (Egel, Lankenau, & Mulkidjanian, 2011), or Nick Lane (Lane, 2015).

Briefly, as an example, one of the most recent suggestions is that the conditions necessary for life, and the origins of the RNA/proteins, could be combined with a prebiotic information system that evolved in a hydrothermal impact crater lake. This could have had the necessary geothermal chemistry, ingredients from meteorites and dry/wet cycling, with UV being important in generating the compounds in space (Chatterjee, 2016; Chatterjee & Yadav, 2019). Thus, although any origin of life theory is still somewhat speculative, it is fair to say that both UV and proton gradients may well have been important. Critically, however it actually started, it is very likely that life has to have its roots in thermodynamics and quantum mechanics, and likely centres on self‐organisation in dissipative structures involving complexity theory, where fluctuations in energy can produce order out of chaos (Pulselli, Simoncini, & Tiezzi, 2009; Trevors, 2011). In short, life can be viewed as a localised ordered system that although it seems to go against the second law of thermodynamics, actually drives the thermodynamic equilibration of the universe. Key in this is its ability to use information (Michel, 2013).

### 2.1 Life as a dissipative structure based on a proton gradient

The concept that life is a dissipative structure, obeying the laws of entropy, underlies one of the strongest theories involving the proton gradient and alkaline geothermal vents, as proposed by Nick Lane in his book ‘The vital question: why is life the way it is?’ (Lane, 2015). Based on this theory, the evolution of photosynthesis would have emerged later and utilised the components of the ETC chain to harness light powered generation of electrons from hydrogen or hydrogen sulphide, in effect, anoxygenic photosynthesis. In this model, UV would have been far too damaging as the emerging cellular life would not have evolved the protective UV systems present in modern phototrophs. However, phototrophy may have then started as organisms started to harness the very low levels of light emitted from the hydrothermal vents themselves—so called ‘geothermal light’. Then, with time, and exposure to light, these systems would have evolved eventually leading to modern oxygenic photosynthesis (Martin, Bryant, & Beatty, 2017). Key in this process, which ultimately led to the great oxidation event, was the ability to utilise water as an electron source and the ability to control excessive electron production under high light conditions, and thus, ROS production, which resulted in the co‐evolution of anti‐oxidant systems (Hamilton, 2019).

In short, one theory on the origins of life suggests it is dissipative and started in alkaline hydrothermal vents and is now dependent on using electrons to drive the formation of a hydrogen gradient, which can either be derived via chemolithotrophy or photolithotrophy, but where UV may well have been too disruptive to begin with. However, once life began to use light, it had to deal with the problem of the generation of ROS.

### 2.2 Life as a structure based on the dissipation of the solar potential

Clearly, one of the most important steps in the evolution of life was storage of information in molecules like DNA or RNA. The origins of the RNA world are still being debated. However, the formation of nucleotides and their polymerisation into molecules like RNA in ‘warm little ponds’ from nucleobases delivered from meteorites due to wet‐dry cycles and the effects of UV, is certainly a strong competing theory to their formation in thermal vents, which may only produce very short nucleotides (Pearce, Pudritz, Semenov, & Henning, 2017). Because of the extreme UV levels on early earth, one school of thought is that the ancestors of RNA nucleobases must have been very good at absorbing this energy and converting it into heat, for instance, barbituric acid and 2,4,6‐triaminopyrimidine (Brister, Pollum, & Crespo‐Hernandez, 2016). In fact it has been observed that many prebiotic chemicals essential for life, in particular aromatic compounds, can also be generated in interstellar/circumstellar environments (Cuadrado et al., 2017; Pearce et al., 2017; Tachibana et al., 2017), suggesting that UV did play a role in creating life's ingredients.

UV may have then continually driven the increasing complexity of molecules by enabling natural selection of photo‐stable nucleotides, perhaps at the interface between thermal vents and sunlight. One hypothesis suggests that the very earliest ‘organisms’ (proto‐life) utilised light as one of the potential source of energy, but as the conditions on the early earth changed and oxygen levels rose, evolution drove the need to utilise other energy sources, such as sodium and proton gradients (Egel et al., 2011; Ranjan, Todd, Sutherland, & Sasselov, 2018).

A similar idea is the ‘UltraViolet and Temperature Assisted mechanism for Reproduction’ (UVTAR) hypothesis, which also describes the origins of RNA and DNA. This theory is based on the idea that the temperature of the early Achaean sea was close to the melting temperature of polymers of these molecules. When exposed to high levels of UV, and other organic molecules and salt, together with the diurnal cycle of heating and cooling, these circumstances would have been ideal to start to select for the most efficient energy dissipating polymers with no need for enzymes. This is because these polymers are more efficient than single nucleotides at taking the energy in a UV photon to the ground state. The temperature of the surface of the early earth, about 3.8 Gyr ago, could have been as high 80°C. The other factor here is the link between information and entropy: as the seas cooled below the annealing temperature of the proto‐DNA/RNA this may have resulted in the selection of molecules that held information that enabled them to dissipate energy more effectively, especially if they combined with molecules such as aromatic ring amino acids, which enhance the breadth of the spectrum of UV absorption. As the seas cooled, combinations of amino acids, which lowered the denaturing temperature still further, would have led to ever greater complexity. It may thus be relevant that purple bacteria, perhaps the most ancient of photosynthetic organisms, employ bacteriochlorophyll in their reaction centres that absorb UV light at 280 and 400 nm, suggesting this could have been a very primitive and early photosynthetic system. This might suggest an alternative explanation of chemoautotrophs; they migrated to thermal vents and evolved to use infrared light first, then started to utilise the energy in these gradients. Today, bacteriorhodopsin, as well as utilising light at 568 nm, can also absorb light at 280 nm by using the aromatic amino acids, tyrosine and tryptophan. Chlorophyll emerged as the atmosphere changed, reducing UV and enhancing longer wavelengths absorption. This hypothesis thus moves the function of life away from ‘self‐perpetuation’ towards the dissipation of the solar flux by its ability to couple it to the earth's water cycle (Michaelian, 2011, 2017).

It is worth emphasising that all these theories point towards a critical role of UV in life's beginnings, in particular, by selecting for molecules that could dissipate its energy.

### 2.3 Cofactors or prefactors: Building on the properties of ancient molecules

The above theories suggest at least three scenarios: metabolism first, with gradual genetic take over; replication first then metabolism; or that replication and metabolism were shared right from the beginning. One observation is that many essential coenzymes display UV absorption spectra, such as the B vitamins (Knak, Regensburger, Maisch, & Baumler, 2014; Monteverde, Gomez‐Consarnau, Suffridge, & Sanudo‐Wilhelmy, 2017). Critically, the central components of the ETC, such as the cytochromes and ubiquinone, as well as the flavins and NAD, all have associated absorption spectra, ranging from the UV to visible. Their spectra change according to their redox status, which certainly in the case of NAD(P)H and FAD^+^, as they fluoresce, has not only become a mechanism to image mitochondria, but also to monitor metabolism (Shuttleworth, 2010). NADH has an absorbance band at 300–350 nm and emits at 430–450 nm, and in water, shows a very high quantum yield in ejecting an electron of 0.46—but a much lower fluorescence quantum yield, which is about 0.02 (Boldridge, Morton, & Scott, 1984). This might indicate that under high UV levels NADH could generate a lot of electrons, which could affect the ETC and lead to the formation of NAD+. Certainly mitochondria are very sensitive to UV light, and can generate ROS when exposed to it (Gniadecki, Thorn, Vicanova, Petersen, & Wulf, 2000).

However, at lower UV intensities, could NAD act as a sunscreen? NADH has an absorption coefficient of about 6,317 L mol^−1^ cm^−1^ at 340 nm (McComb, Bond, Burnett, Keech, & Bowers Jr., 1976), which although at the lower end, is in the same range as commercially available sunscreens that exhibit absorption coefficients from 4,300 for homosalate X (based on salicylic acid), to over 20,000 for some of the cinnamates (Shaath, 2010). Moreover, both NADH and NAD+ also absorb at 260 nm, so although NAD+ is not fluorescent, it can still absorb UV. Thus, although both of forms of NAD could act as sunscreens, it could also hint at how UV may have played a role in its origins as a key component of redox and energy metabolism; these factors were probably inter‐linked.

If these observations are combined with the UVTAR theory, in particular, if very early organisms could use UV for photosynthesis, which some still apparently can do (Haas et al., 2018), then the suggestion that mitochondria may have descended from a purple non‐sulphur bacterium, where the cristae may have been part of the light harvesting mechanism (Munoz‐Gomez, Wideman, Roger, & Slamovits, 2017), is interesting. It has been suggested that the core components of the ETC, the cytochrome *bc* complexes, may have originally evolved as part of a light harvesting system, but switched function as oxygen levels increased (Dibrova, Shalaeva, Galperin, & Mulkidjanian, 2017). NAD could have played a number of roles in these circumstances.

Of possible relevance here is the importance of tryptophan, a UV absorbing molecule that is also degraded by UV, but can also be generated by abiotic chemistry, and its importance in the extant NAD+ synthesis pathway. It could be argued that the photo‐chemical properties of tryptophan have become imprinted by the evolution of enzymes, in effect, accretion. This would suggest that ancient stress resistance pathways, such as those involving the sirtuins, could have originally evolved in response to UV. Not only are sirtuins an ancient central adaptive pathway to oxidative stress involving mitochondrial function that modulate ageing (Greiss & Gartner, 2009; Singh et al., 2017), but they are also involved in UV‐induced DNA repair, and require NAD+ as a cofactor (Fan & Luo, 2010). Interestingly, as it is now thought that NADH could well have been generated by prebiotic chemistry, as could peptides containing cysteine residues. These could have come together in an Fe‐S cluster in proto‐cells to generate a pH gradient; this has been demonstrated in vesicles (Bonfio et al., 2018). Critically, it has also been suggested that UV was key in the prebiotic synthesis of Fe‐S clusters, which are central to the ETC (Bonfio et al., 2017).

It is thus appears likely that life largely utilises an ETC that relies on compounds that have structures that enable them to both absorb UV and act in redox reactions to maintain a proton gradient. These UV absorbing compounds are also key in information storage, such as DNA and RNA, but also share structural similarities to the main energy currency in the cell, ATP, as well as NAD. Thus the natural selection of relatively photo‐stable and redox‐stable molecules does seem to have been key in the origins of life, as is the smooth flow of electrons when there are large variations of energy input.

It may therefore be significant that the ETC could well be utilising electron tunnelling, which could be key in how it tunes itself to changes in the environment (de Vries, Dorner, Strampraad, & Friedrich, 2015; Moser, Farid, Chobot, & Dutton, 2006), suggesting that hormesis could also be viewed as a way of maintaining significant quantum tunnelling in mitochondria (Nunn, Guy, & Bell, 2016). Indeed, it has been suggested that proteins have evolved to exist at quantum criticality (Vattay, Salahub, Csabai, Nassimi, & Kaufmann, 2015), as exemplified by the environment‐assisted quantum transport (ENAQT) theory (Zerah‐Harush & Dubi, 2018). This suggests that biology may have selected for quantum transport mechanisms to enhance the efficiency and *robustness* of energy transport in biological systems; key in this is that proteins may enhance the innate exciton transfer ability of chromophores by ensuring long lived ‘coherence’ by a form of vibronic resonance (Rathbone et al., 2018a, 2018b). This coherence is mostly discussed in relation to light harvesting systems, but may well be more broadly applicable to many biological systems to maintain robustness (Scholes et al., 2017) when electrons are also considered. In biological terms, cofactors are often described as enabling the functional properties of proteins because they enable redox activity and electron transport, and usually take the form of metal complexes, cytochromes or flavins. It is thus relevant that researchers looking into protein bioelectronics discuss ‘doping’ proteins to enhance electron transport with compounds such as hemin (Bostick et al., 2018).

In summary, it could thus be speculated that life is built on chromophoric molecules whose inherent light absorbing and electron transfer characteristics have been amplified by proteins to ensure robust energy dissipation—possibly by enhancing fundamental quantum principles. So the reality is that ‘cofactors’ are probably much more likely to be ‘prefactors’, as they were simple molecules that were generated abiotically, and proteins evolved around them and quite possibly, through a process like ENAQT, enhanced the ability of these systems to extend quantum effects further into the mesoscopic realm.

## 3 SECONDARY METABOLITES AND ENERGY DISSIPATION

Photosynthesis could be viewed as a way of efficiently dissipating the solar potential in a chromophore landscape to drive metabolism (Ho et al., 2017; Thyrhaug et al., 2018). As secondary plant compounds may have originally evolved as UV ‘sunscreens’ (Cheynier, Comte, Davies, Lattanzio, & Martens, 2013), and many of these compounds do have medicinal activity (Baran et al., 2011; Bowd, Byrom, Hudson, & Turnbull, 1971; Diaz, Freile, & Gutierrez, 2009; Lopez‐Nicolas & Garcia‐Carmona, 2008; Mondal, Ghosh, & Moulik, 2016), do they modulate dissipation as a means to maintain homeostasis?

In plants, mitochondrial function is pivotal to efficient photosynthesis (Araujo, Nunes‐Nesi, & Fernie, 2014) and can be stimulated under stress, for instance, when under high light irradiation, where they can absorb excessive electrons (Jacoby et al., 2012). Certainly, it has been known for some time that plant mitochondria are central to controlling plant cell redox in response to stress (Noctor, De, & Foyer, 2007). Likewise, mitochondria from animals not only actively produce hydrogen peroxide, but they can remove it, so acting as net consumers, or producers, of ROS (in the form of hydrogen peroxide) depending on circumstances (Drechsel & Patel, 2010; Kamunde, Sharaf, & MacDonald, 2018).

So, in a way, mitochondria can also dissipate energy to prevent damage, much in the same way as a radiator in a car can prevent over‐heating damage. So has evolution built upon the basic ability of these plant chromophores to dissipate energy by adopting this to control more elaborate energy dissipation mechanisms, such as the ability of mitochondria to dissipate excess energy? If they have, through evolution, they would thus appear to control many integrated systems, for instance, calcium signalling and uncoupling of the proton gradient. As many of these secondary metabolites also have anti‐bacterial actions, and mitochondria evolved from a prokaryote, could the dissipation idea also be applied as one of the ways these compounds could also help the plant, or animals, in pathogen defence by invoking too much dissipation?

### 3.1 Dealing with excess electrons; dissipation and uncoupling

There is a basic structural relationship between the ability to act in redox reactions, and the ability to absorb light, which is associated with conjugated structures containing multiply double bonds. One of the most obvious examples of this are the flavonoids, which, although they can act as sunscreens in the plant, are also anti‐oxidants that have evolved to also play a generalised role in protecting against stress (Brunetti et al., 2018). Ideal sunscreen molecules contain chromophores that have the ability to absorb and then safely dissipate photon‐derived energy without rapidly breaking down and so potentially initiating a damaging chemical reaction. This energy can be released as a longer wavelength photon, for instance by fluorescence, but can also be transferred to other molecules by energy transfer, structural conformational changes, as well as proton‐coupled electron transfer (PCET): the ability to dissipate this energy and absorb across a greater number of wavelengths is generally enhanced by large conjugated systems, but also the local environment (Baker, Marchetti, Karsili, Stavros, & Ashfold, 2017).

Critically, excessive photorespiration can generate a high flow of electrons into mitochondria, which can also help to protect the plant in high light conditions; uncoupling proteins (UCPs) and alternate oxidases (AOX) seem to play a role in this, as well as other organelles, such as peroxisomes (Sunil, Talla, Aswani, & Raghavendra, 2013; Sweetlove et al., 2006). Mitochondria in animals can also help cells survive endoplasmic reticulum (ER) stress by absorbing excessive ROS via enhanced respiration (Knupp, Arvan, & Chang, 2018). Simply put, mitochondria can act as electron sinks—a kind of a cellular equivalent of the energy dissipating function of the radiator in a motor vehicle. It is also true that mitochondria are also a primary source of ROS, but this can be part of the key signalling adaptive process (Ristow & Zarse, 2010), which hints at a very tight coupling between these processes. But how do mitochondria, and motor vehicles for that matter, deal with the excess energy if they cannot readily turn it into something useful? They ‘uncouple’ the energy generation and utilisation process, and generate heat. It also enables the cell (or car) to ‘tick over’, so it is always ready for anything. In fact the extra heat is actually useful, for instance, for endothermy, and for keeping the occupants of a car warm. Of course, it is also a fundamentally dissipative process, which enables structure to be maintained.

Uncoupling is a term used in biology to describe the process by which cells ‘uncouple’ the production of the energy‐rich molecule, ATP, from proton gradients by effectively short‐circuiting the system, resulting in an apparent futile cycle that generates heat. Indeed, there is evidence that electron transport through the ETC does increase mitochondrial temperature, and uncoupling agents can increase the mitochondrial temperature by 12°C or more (Chretien et al., 2018). In light of this data, it may be no coincidence that the parallel arrays of cristae membranes within mitochondria do look a little like radiators (Lane, 2018). If the dissipative theory is correct, it is unsurprising that both prokaryotes and eukaryotes demonstrate uncoupling; depending on the cell type, between 20 and 50% of energy is dissipated—and many systems have evolved to control it, in particular, the UCPs (Russell, 2007; Stuart, Brindle, Harper, & Brand, 1999). UCPs are certainly common to plants and animals, and significantly, evidence continues to suggest that one of their main functions is to control ROS (Mailloux & Harper, 2011; Nogueira, Borecky, Vercesi, & Arruda, 2005; Woyda‐Ploszczyca & Jarmuszkiewicz, 2016).

### 3.2 Phenol—A core moiety found in many plant compounds

A common ‘core’ moiety found in all life is the phenol moiety, for instance in the amino acids tyrosine and phenylalanine, and in scytonemin—a key UV protectant produced by cyanobacteria, suggesting this structure was adopted early in evolution (Cockell & Knowland, 1999). The phenol structure can be created by basic chemistry and is therefore likely to have been present on the early earth before life evolved (Egel et al., 2011), suggesting it could have been a key part of the ‘tool kit’ required to get life going. The phenol structure is fluorescent, displaying maximum absorbance around 270 nm, and emitting at 296 nm (Tchaikovskaya et al., 2000), which not only means that many plant phenolic compounds are able to absorb light, but they can demonstrate redox cycling, acting as both direct anti‐oxidants and oxidants depending on the redox potential and the existence of other electron donors/acceptors (Perron, Garcia, Pinzon, Chaur, & Brumaghim, 2011).

These properties are explained by the fact that phenol, and thus related plant compounds, contain a π‐electron system that enables them to absorb highly energetic UV light by exploiting basic quantum principles and dissipating excessive energy via resonance; lengthening the double bond system and increasing the complexity of these molecules increases the wavelength they can absorb (Cockell & Knowland, 1999). This process is perhaps clearest in photosynthesis, where carotenoid molecules, which although they do not have aromatic structures, do have large conjugated double bond systems, which can quench chlorophyll triplet states that might otherwise lead to singlet oxygen (Ho et al., 2017). Another property of many of these compounds, in particular aromatic compounds like benzene and their derivatives, is that the delocalisation enhances their stability, and enables them to take part in redox reactions without being destroyed (for a more detailed description of the physical chemistry and fluorescence properties of these compounds, see [Atkins & de Paula, 2014; Lakowicz, 2006]).

It would therefore seem, in reference to the previous examples, that biology has exploited under some circumstances the potential resonant quantum properties of double bond systems in various structures to absorb, and dissipate, the energy from both photons and electrons. In short, the double bond structure, in the right molecule, enables them to both absorb photons and take part in redox reactions.

Finally, phenol is an uncoupling agent and is therefore a central structure in many powerful mitochondrial uncoupling agents that ‘uncouple’ the proton gradient from ATP production (Terada, 1990). Originally it was thought that the mechanism was based around direct bulk proton transport across the membrane, but there are other theories suggesting it may involve ATPase (Nath, 2018). This might suggest that the uncoupling process invoked by these compounds is more controlled, or viewed from the prefactor concept, the uncoupling process evolved around the compound itself. This is of course dissipation.

### 3.3 Salicylic acid is an uncoupling agent

As an example, salicylic acid is particularly interesting, as it has a very long history of use as a medicine and contains the phenolic group. Salicylic acid is a plant stress response hormone and has been shown to upregulate the AOX and other NADH dehydrogenases, which can divert electrons out of the ETC and can uncouple ATP production. Critically, not only can it directly uncouple mitochondria at lower concentrations (<100 μM) and inhibit complex III of the ETC at higher concentrations (0.5–2.5 mM) (de Souza et al., 2011; Norman, Howell, Millar, Whelan, & Day, 2004), but when esterified to fatty acids, it is also a very good sunscreen, found in many proprietary sun creams (Kapadia et al., 2013). It is fluorescent, especially in detergents, with two excitation (absorption) peaks at 275 and 322 nm, emitting at 408 nm (Karim, Lee, Kim, Bae, & Lee, 2006). While mild uncoupling can also be mechanism to reduce ROS and plays a key role in mitochondrial redox modulation (Jezek, Holendova, Garlid, & Jaburek, 2018), and can even slow the ageing process (Barros, Bandy, Tahara, & Kowaltowski, 2004), it can also generate heat in thermogenic plants, such as the Arum Lily (Raskin, Turner, & Melander, 1989). In fact several types of plant display homoeothermy and can generate heat in a number of ways, which are mainly centred around the mitochondrial respiratory chain, such as the activation of AOX and UCPs, particularly in the inflorescences and flowers: it is thought this serves a variety of functions ranging from volatilising scents, growth in cold climates and enabling germination and pollen tube growth (Zhu et al., 2011). In some circumstances, uncoupling is emerging as an anti‐inflammatory mechanism in animals—as it can stop mitochondria producing excessive ROS (Du et al., 2016; Emre & Nubel, 2010). Thus salicylic acid is an example of a chromophoric molecule that that can help a plant survive stress, and can act as an uncoupling agent and a medicine.

### 3.4 Many plant compounds target the mitochondrion

Part of the mode of action of many plant compounds, at least in animals, seems to involve upregulation of mitochondrial function as part of an anti‐oxidant mechanism (Stevenson, 2012), as well as displaying ROS‐scavenging independent actions (Sandoval‐Acuna, Ferreira, & Speisky, 2014). Indeed, several well‐known medicinal plant compounds can directly modulate mitochondrial function, including epigallocatechin gallate (EGCG), resveratrol (Battaglia, Salvi, & Toninello, 2005; Demos, Woolwine, Wilson, & McMillan, 1975; Oliveira, Nabavi, Daglia, Rastrelli, & Nabavi, 2016; Pereira et al., 2007; Ravanel, Tissut, & Douce, 1982; Usta et al., 2009; van Ginkel et al., 2007; Xie, Bezard, & Zhao, 2005), tetrahydrocannabinol (THC) (Athanasiou et al., 2007; Bartova & Birmingham, 1976; Fisar, Singh, & Hroudova, 2014; Mahoney & Harris, 1972) and CBD (Fisar et al., 2014; Mato, Victoria Sanchez‐Gomez, & Matute, 2010; Olivas‐Aguirre et al., 2019; Rimmerman et al., 2013; Ryan, Drysdale, Lafourcade, Pertwee, & Platt, 2009), as well as salicylic acid (de Souza et al., 2011; Gordon et al., 2002; Norman et al., 2004; Nulton‐Persson, Szweda, & Sadek, 2004; Vlot, Dempsey, & Klessig, 2009) and curcumin (Lim, Lim, & Wong, 2009; Trujillo et al., 2014). It also seems that some of the more ancient sunscreens, such as scytonemin can also modulate mitochondria (Itoh et al., 2013).

In terms of mitochondrial targets, several plant compounds can modulate the voltage dependant anion channel 1 (VDAC1), the main channel in the outer mitochondrial membrane, including, salicylic acid, curcumin, and CBD (Alfonso et al., 2014; Gorlach et al., 2015; Guzman et al., 2006; Rimmerman et al., 2013; Tewari et al., 2015; Tewari, Majumdar, Vallabhaneni, & Bera, 2017; Torres et al., 2011; Vara et al., 2011), while many interact with components of the ETC, or F0F1‐ATPase/ATP synthase, and some can affect both (Bohmont, Aaronson, Mann, & Pardini, 1987). For instance, quercetin can modulate complex‐1 of the ETC at low concentrations (<10 μM), reducing mitochondrial hydrogen peroxide production and inhibiting apoptosis (Lagoa, Graziani, Lopez‐Sanchez, Garcia‐Martinez, & Gutierrez‐Merino, 2011). Similarly, berberine can also inhibit complex 1 (Turner et al., 2008), as can CBD, THC and resveratrol (Athanasiou et al., 2007; Fisar et al., 2014; Zini, Morin, Bertelli, Bertelli, & Tillement, 1999). Resveratrol, EGCG, curcumin and quercetin have also been shown to have effects on F0F1‐ATPase/ATP synthase (Zheng & Ramirez, 2000). Interestingly, a derivative of curcumin, J147, has been shown to partially inhibit ATP synthase, leading to an increase in mitochondrial potential and superoxide, and the activation of calcium/calmodulin‐dependent protein kinase kinase β (CamKK2) via an increase in intracellular calcium, leading to the activation of AMPK and inhibition of mTOR. The net result appears to be neuroprotective, attenuating age associated decline and extending lifespan (Goldberg et al., 2018). It is also clear that many plant compounds, such as the theaflavins, also inhibit bacterial ATP synthase and ETC components, as well as inhibiting the same targets in mitochondria—apparently without inducing the production of superoxide (Li, Vik, & Tu, 2012).

Critically, the effects of resveratrol on complex 1 have been shown to be biphasic (Madreiter‐Sokolowski, Sokolowski, & Graier, 2017). This is particularly interesting as evidence now indicates that the ROS generating component of complex 1 itself, at least in animals, can be deliberately degraded in depolarised mitochondria to avoid mitophagy (Pryde, Taanman, & Schapira, 2016).This suggests that these plant compounds, at lower doses, can also act to reduce ROS production—perhaps by inducing the degradation of the ROS producing component of complex 1. Data now shows that specific inhibitors of complex 1 can reduce reverse electron transport, and improve the ability of mitochondria to absorb ROS (Kamunde et al., 2018). In contrast, it seems that salicylic acid, at higher concentrations (>50 μM) inhibits complex III and respiration in plant mitochondria, which generates a ROS signal that can upregulate the production of the AOX, which is part of a system to help reduce mitochondrial ROS production in plants (Nie, Yue, Zhou, & Xing, 2015). It is also worth noting that quercetin at higher concentrations (50 μM) increases intracellular calcium levels and lower NAD(P)H concentration—inducing apoptosis (Baran et al., 2011).

**Figure 1:**
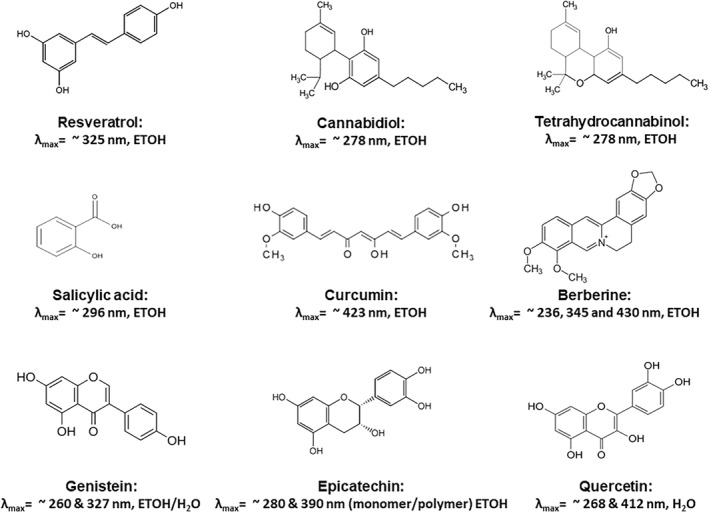
Some medicinal plant compounds that affect mitochondria and their absorption spectra

Thus, overall, the evidence is that many plant secondary metabolites do, indeed, modulate mitochondrial function, which does support their role in stress adaptation. Figure 1 depicts some of the key plant compounds known to have medicinal activity, which also modulate mitochondrial function.

### 3.5 Anti‐bacterial actions of plant compounds—Dissipating to death?

Interestingly, although a great deal of research has gone into studying the anti‐bacterial mode of action of many plant compounds, there is still uncertainty as to the precise underlying mechanism for many of them. For instance, it has been suggested that aggregation, inhibition of energy production, membrane disruption, enzyme/receptor/channel modification, inhibition of quorum sensing are all involved—but many do seem to synergise with more conventional antibiotics (Cushnie & Lamb, 2011; Daglia, 2012).

Many bacteria can ‘energy spill’ to enable survival—often by futile cycling of ions through their membranes (Russell, 2007). However, too much dissipation can cause gradient collapse, loss of structure and death. Therefore the finding that phenol, and many compounds containing this chemical moiety can act as mild protonophoric uncoupling agents (Terada, 1990), is noteworthy. Phenol is well known to have anti‐pathogenic functions, which was of course championed by Joseph Lister as ‘carbolic acid’ to sterilise surgical instruments and wounds in the 19th century. In fact phenol‐containing molecules, such as the phytocannabinoids, display potent anti‐microbial activity; structure–activity studies indeed show that the phenol moiety is important in determining these compounds anti‐bacterial activity (Appendino et al., 2008). Certainly, other phenolic containing compounds, including salicylic acid and other non‐steroidal anti‐inflammatory compounds, show a wide spectrum of anti‐bacterial activity, and are being investigated for use in conditions like bacterial endocarditis, urinary tract infections and diabetic foot infection (Ahmed et al., 2017; Akhter, Baqai, & Aziz, 2010; Kupferwasser et al., 1999; Schoergenhofer, Schwameis, Lagler, & Jilma, 2016). Curcumin also has anti‐bacterial properties, although these are limited by absorption issues, but various formulations can improve this, and it is being looked at as a lead compound to develop more useful analogues (Liang et al., 2008; Xie et al., 2015). Resveratrol also has anti‐bacterial properties (Bostanghadiri et al., 2017).

Although bacteria do not have VDAC channels, they do have porins, which utilise a similar β‐barrel structure (Zeth, 2010), so it is possible that plant compounds can also modulate these. Unfortunately, the role of calcium homeostasis is still not well understood in bacteria, but they do contain all the necessary channels, transporters and binding proteins, including calcium‐dependent ATPases that are key in exporting it out of the cell (Dominguez, Guragain, & Patrauchan, 2015).

As evolution tends to ‘tinker’ and find new useful functions for molecules, it is entirely possible a compound can end up modulating many different pathways (Jacob, 1977). One only has to look at calcium, ROS, or compounds such as NAD, to see this process at work. This must be true for compounds similar to CBD or resveratrol which may well have started out as protection/signalling moieties, but if they could also inhibit bacterial growth, evolution would have adopted this. For instance the acid forms of CBD and THC (CBDA and THCA, which are the predominate form in the plant), can induce cell death in cannabis leaves at concentrations of 100 μM or more (Morimoto et al., 2007; Shoyama, Sugawa, Tanaka, & Morimoto, 2008). The plant stores most of these compounds in trichomes just above the leaves, so at least from a pathogen point of view, releasing very high concentrations onto a leaf would not only potentially kill a pathogen, but also induce death of the leaf cell itself, which is also an anti‐pathogen mechanism. Lower concentrations at a distance could then potentially prepare other cells by acting as a signal. It could also be argued that at high concentrations they could also protect the leaf and sexual organs from excessive UV—which has been a long held theory (Pate, 1983).

## 4 A WINDOW ON SECONDARY METABOLITE ACTION: MITOCHONDRIA AND CALCIUM

Mitochondrial dysfunction is a common finding in many diseases and the general ageing process (Ferrucci & Fabbri, 2018; Lane, 2003). In fact, it is becoming clear that mitochondria, via several signalling mechanisms, can control the aging process, either speeding it up or slowing it down (Dakik, Medkour, Mohammad, & Titorenko, 2019). This is not surprising as not only are mitochondria key in releasing energy, they also play a central role in environmental sensing, signalling, cell fate and immunity, both in animals and plants (Colombatti, Gonzalez, & Welchen, 2014; Lane, 2005; Schwarzlander & Finkemeier, 2013; West, Shadel, & Ghosh, 2011).

It seems that a key underlying factor that likely determines lifespan, and the propensity to develop pathology, is related to how well mitochondria control their release of ROS, which in turn appears to be related to how much spare capacity they have in terms of electron flow. Organisms, such as birds, have very high aerobic capacity, which even with a very high metabolic rate, is associated with minimal reduction of the ETC, a reduced need for anti‐oxidants and a sensitive ROS‐base retrograde signalling system. This seems to partly explain why a pigeon lives much longer than a comparable sized mammal, such as a rat. It also explains why ‘anti‐oxidants’ do not extend life. Critically, having a lot of spare ETC capacity means that the system can easily continue to uncouple, so reducing ROS generation, while maintaining energy levels (Lane, 2005).

But there is another factor worth considering; mitochondria are also central to calcium metabolism (Szabadkai & Duchen, 2008): healthy mitochondria equal healthy calcium dynamics. It is therefore interesting that many plant compounds, such as salicylic acid, CBD, resveratrol and quercetin can modulate intracellular calcium in animals (Bardy et al., 2013; Ibeas Bih et al., 2015; McCalley, Kaja, Payne, & Koulen, 2014; Ryan et al., 2009; Suzuki, Inoue, & Ra, 2010).

### 4.1 Calcium signalling and disease

Calcium is now thought to have been central in the evolution of life itself (Kazmierczak et al., 2013) and its role in signalling can now be traced from prokaryotes to modern organisms (Cai, Wang, Patel, & Clapham, 2015). Critically, because of its role in energy generation—negative and positive, it is thought that the very earliest steps of endosymbiosis that led to the modern eukaryotic cell meant that the α‐proteobacterium that became the mitochondrion would have been pivotal to the evolution of calcium signalling in these cells (Blackstone, 2015). Today, calcium signalling therefore controls every aspect of cell function, from energy production to growth and death. The signalling process is highly compartmentalised, for instance, directing calcium at the right concentrations towards the cytosol and nucleus can stimulate growth and inflammation, while enhancing mitochondrial uptake can stimulate the production of ATP and ROS, but also lead to cell death. In fact, it is now clear this is one of the adaptations that happens in cancer: it seems that the calcium signalling is shifted towards growth and away from death (Monteith, Prevarskaya, & Roberts‐Thomson, 2017). Key components involved in this redirection from cell death to growth are mitochondria‐associated membranes (MAMs), of which VDAC1 is a central protein (Danese et al., 2017). It is also becoming apparent that disrupted calcium homeostasis plays a role in metabolic diseases such as type 2 diabetes associated with obesity, which are also associated with mitochondrial dysfunction and inflammation (Arruda & Hotamisligil, 2015).

Calcium signalling is just as important in plants as it is in animals (Kleist & Luan, 2016), for instance, it plays important roles in plant immunity (Yuan, Jauregui, Du, Tanaka, & Poovaiah, 2017) and photosynthesis (Hochmal, Schulze, Trompelt, & Hippler, 2015). Critically, as plants also suffer from cancer, it has been suggested that many of these secondary plant metabolites could help protect the plant (Rasouli, Farzaei, Mansouri, Mohammadzadeh, & Khodarahmi, 2016). It is therefore relevant that salicylic acid, genistein, resveratrol, curcumin, berberine and CBD show promise as anti‐cancer agents in animals (Adiwidjaja, McLachlan, & Boddy, 2017; Alfonso et al., 2014; Massi, Solinas, Cinquina, & Parolaro, 2013; Ortiz, Lombardi, Tillhon, & Scovassi, 2014; Pervaiz & Holme, 2009; Spagnuolo et al., 2015). In fact over 3,000 plant species seem to have anti‐cancer effects, with many involving mitochondrial function in their actions (Gali‐Muhtasib, Hmadi, Kareh, Tohme, & Darwiche, 2015). Indeed, polyphenols could be viewed as mitochondrially‐targeted anticancer drugs (Gorlach et al., 2015). It is thus likely that both in the plant, and in animals, these compounds may modulate calcium homeostasis as a protective response. At the present time, most of the calcium data refers to the effects of these compounds in animals, but it would be interesting to speculate that many more of them may also control calcium dynamics in plants.

### 4.2 Uncoupling and calcium

Critically, it seems that UCPs can also modulate calcium flux (Motloch et al., 2016), and that UCP‐independent calcium cycling is another dissipative process, generating heat (Ikeda et al., 2017), but too much uncoupling can also cause mitochondrial failure, alter calcium signalling and increase ROS production. This is ably demonstrated by uncoupling agents such as carbonyl cyanide 4‐(trifluoromethoxy) phenylhydrazone (CCCP), but in particular, by the well‐known phenolic anti‐bacterial agent, triclosan (Weatherly et al., 2018). As the mitochondrial membrane potential is a determinate of calcium flow, and the movement of calcium itself also determines the mitochondrial membrane potential as it is charged, then there is a fundamental relationship between calcium and mitochondrial function, and thus, of course, ROS (Brookes, Yoon, Robotham, Anders, & Sheu, 2004). Hence, although uncoupling, and thus energy dissipation often refers to a short circuit in the proton gradient, it can also involve changes in calcium flux, as calcium ions are also charged and their differential concentration across a membrane represents a store of energy.

### 4.3 UV and calcium

Although cholesterol is mainly found in eukaryotes, its precursors in prokaryotes, the terpenoids, are essential in prokaryotic membrane structure (Ourisson & Nakatani, 1994), and absorb in the UV range (Walker & Hawkins, 1952). In fact, cholesterol‐like molecules may have started out as a sunscreens, but after photoactivation, not only could they have then signalled a primitive seaborne organism to move, but may have also led to the use of calcium as a cytoskeleton (Wacker & Holick, 2013). Today, other than its key role in calcium metabolism, vitamin D has been found to modulate mitochondrial function, increasing respiration, and is important in muscle function (Ryan et al., 2016). Critically, it has been found to interact with the sirtuins as part of a mechanism to enhance resistance to oxidative stress (Manna, Achari, & Jain, 2017; Sabir et al., 2017).

Exposure of eukaryotic cells to UVB, in particular, at 340 nm, induces calcium influx (Mendez & Penner, 1998). Prokaryotes also rely on the proton motive force to maintain a membrane gradient, which is essential for controlling calcium flux; inhibition rapidly results in the loss of ability to pump calcium out of the cell and light at 360 nm has been shown to inhibit quinone function, an essential part of the ETC, which can inhibit the maintenance of this gradient (Deves & Brodie, 1981). Given the importance of NADH in all life, and the fact that it absorbs at 340 nm (and at 260 nm, critically, NAD^+^ also absorbs at 260 nm), this may not be a coincidence.

In effect, although UV can affect many systems, its ability to modulate the ETC may be more important than previously thought, which must be tightly integrated with calcium signalling. Certainly, NAD^+^ is now viewed both as a central signalling and metabolic molecule, and in particular, its metabolites, such as nicotinic acid adenine dinucleotide phosphate, are some of the most powerful known calcium releasing agents (Guse, 2015).

Quite apart from the role of NAD^+^ in controlling sirtuin function, and potentially being able to absorb UV, and thus act as a sunscreen, it also seems that it plays a central role in calcium signalling and bioenergetics, which might suggest that compounds that evolved to protect against UV, including NADH itself, would also be able to modulate calcium flux. Thus, as we have suggested, if NADH can act as a sunscreen, once it has been converted to NAD^+^, it can start to initiate adaption—this would clearly be an UV intensity‐driven process. But it could also be said that it could, by modulating calcium flux, initiate another form of futile cycling that maintains the dissipative structure. In effect, if the capacity of NADH to act as a sunscreen is exceeded, evolution has selected for other ways for the energy to be dissipated.

### 4.4 Mitochondrial dynamics and calcium

Prokaryotes have existed as complex communities for billions of years, demonstrating cooperation, different subtypes, sessile and motile forms, dying for the greater good, and competition with others to enable survival of the species, as well as cooperation with other species (Be'er et al., 2009; Ben‐Jacob, Cohen, & Gutnick, 1998; McGlynn, Chadwick, Kempes, & Orphan, 2015). Bacteria are clearly adversely affected by UV, and it has been shown that UV can induce cell death, but interestingly, before dying, some species can release vesicles that seem to be protective (Gamalier, Silva, Zarantonello, Dias, & Melo, 2017), while others release UV absorbing compounds, such as scytonemin or mycosporine (Ehling‐Schulz, Bilger, & Scherer, 1997). Hence mitochondria, because of their ancestry display many of the same characteristics; they are both modulated by, and modulate, calcium signalling, which is intimately related to mitochondrial dynamics (Bravo‐Sagua et al., 2017; Szabadkai et al., 2006).

UV can induce mitochondrial uptake of calcium that can lead to apoptosis (Lao & Chang, 2008), which is preceded by mitochondrial fission (Zhang, Liu, Wu, & Xing, 2016). Thus mitochondrial dynamics does provide a good readout of cellular status in health and disease: both fusion and fission are important. For instance, fusion can increase stress resistance, enhance energy production and resistance to mitophagy and cell death, and so is an immediate adaptive response. However, fission is also necessary to enable damaged mitochondria to be removed by mitophagy to maintain a healthy pool of mitochondria, or eventually, it can lead to apoptosis (Sharma, Smith, Yao, & Mair, 2019). Critically, acute inflammation seems to drive mitochondrial dysfunction and fission, which is associated with enhanced ROS production and a switch to glycolysis, but with enhanced autophagy, and then a time dependent activation of mitochondrial biogenesis and re‐fusion of the mitochondrial networks that is associated with resolution (Motori et al., 2013). However, in chronic inflammatory conditions, such as in cancer cachexia, the re‐establishment of functional mitochondria to prevent loss of muscle mass does not happen, but can be partially reversed by exercise, which is a powerful stimulus for mitochondrial biogenesis (VanderVeen, Fix, & Carson, 2017).

Key in explaining this is that mitochondria control cytosolic oscillatory calcium signalling, which in turn, can, for instance, control the activation of two key inflammatory transcription factors, nuclear factor of activated T‐cells (NFAT) and nuclear factor kappa‐light‐chain‐enhancer of activated B cells (NF‐kB). In cancer, which is associated with the activation of inflammatory pathways, mitochondrial uptake of calcium is inhibited, leading to activation of NFAT and NF‐kB (Uzhachenko, Shanker, Yarbrough, & Ivanova, 2015). It is thus relevant that at least in cancer cells, mitochondrial fission and calcium signalling, involving ROS, form a positive feedback loop that enhances autophagy (Huang et al., 2017). This would suggest that agents that could alter the flow of calcium in different parts of the cell could be effective in stopping this feedback loop.

**Figure 2:**
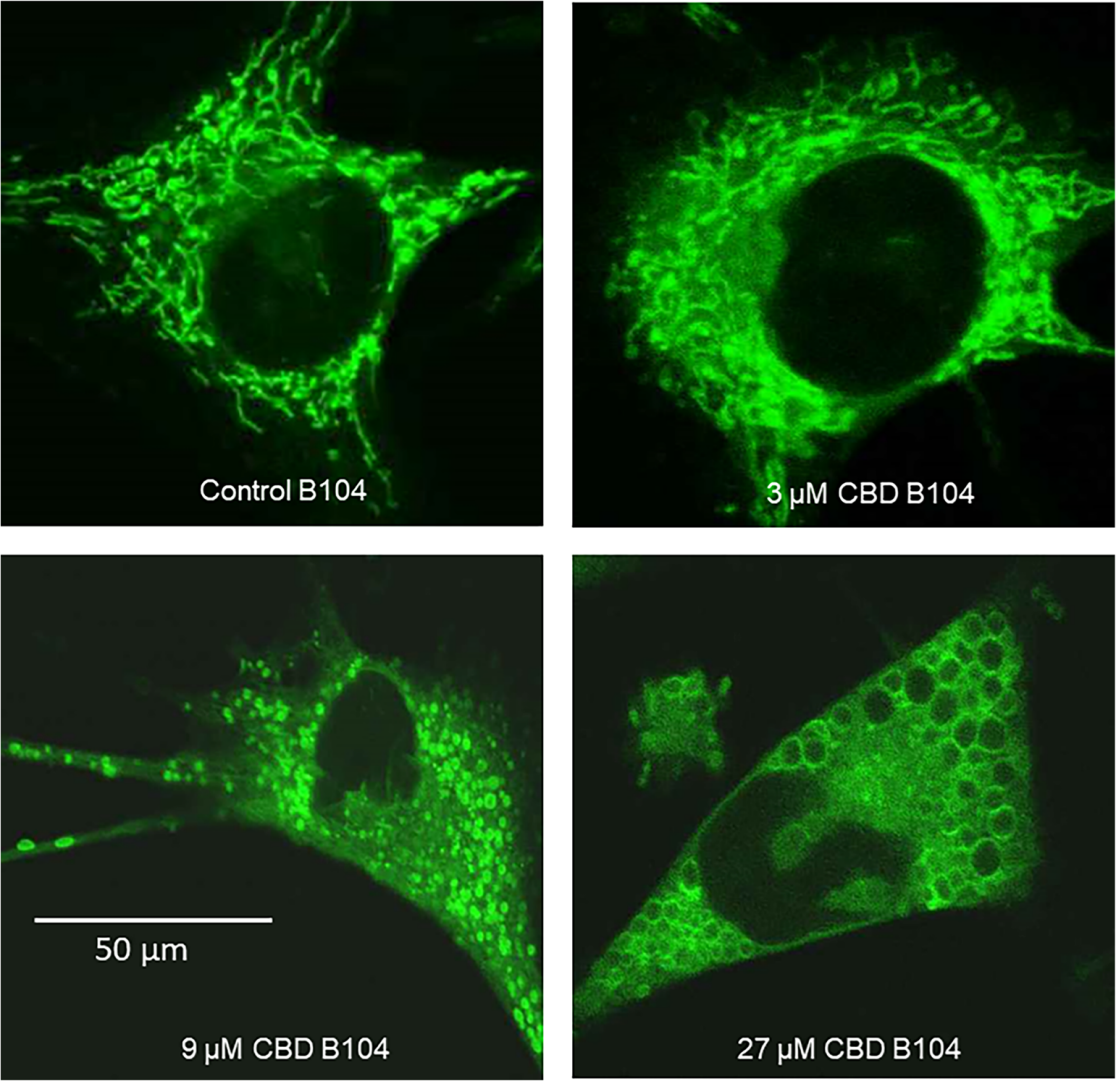
Effects of CBD on mitochondrial structure in animal cells. B104 are a mouse neuroblastomacell line. Mitochondria were visualised using Mitotracker© green after 3 hours. The images are typical cells taken at high magnification using an oil lens. At 3 µM CBD induces formation of small loop structures, with some evidence of fusion and the formation of donuts, characteristic of an initial stress response. At 9 µM it induces fission, with evidence of some mild mitochondrial swelling. At 27 µM it seems to be inducing massive mitochondrial swelling. This can be partly explained by modulation of calcium flux, and in part, could be related to the effects of CBD on VDAC1. This is data taken from the authors published abstract (referenced in main text)

As would be expected, many plant compounds that modulate mitochondrial function and calcium signalling can also modulate mitochondrial dynamics. In this regard, resveratrol has been shown to modulate mitochondrial dynamics in several cell types—inducing both fusion and fission (Li et al., 2016; Ren et al., 2017; Robb et al., 2017). We have also shown that CBD can induce mitochondrial morphological changes in a mammalian cell line, ranging from possible fusion, to fission to ‘donuts’ and eventually, full scale swelling (Figure 2) (Nunn, Henley, Brody, & Bell, 2013). Rimmerman and colleagues have proposed that CBD can induce mitochondrial swelling in a process involving VDAC1 and activation of the mitochondrial permeability transition pore (MPTP) (Rimmerman et al., 2013). More recently, Olivas‐Aguirre and colleagues have also shown that CBD directly modulates mitochondrial calcium in lymphoblastic leukemic cells, showing clear dose related effects, ranging from stimulation of growth to induction of apoptosis (Olivas‐Aguirre et al., 2019). This would support the importance of the MAM in this process, as VDAC is part of the MAM structure.

These changes are very reminiscent of mitochondrial stress adaptation to other modulators, including hypoxia and the uncoupling agent CCCP, as well as valinomycin. During this process, it seems that mild mitochondrial swelling, which can be related to MPTP opening and/or potassium influx, causes detachment of the mitochondria from the cytoskeleton and end‐to‐end autofusion, resulting in toroidal shaped ‘donuts’, which seem to better resist swelling and can recover (Liu & Hajnoczky, 2011). Interestingly, further evidence that a sudden, but temporary influx of calcium into a cell leading to mitochondrial swelling does not always lead to cell death may occur during ‘pseudoapoptosis’, which is also associated with the temporary formation of toroidal mitochondria (Mackenzie, Young, Adinolfi, & Surprenant, 2005). Other research groups have also shown the extent of mitochondrial resilience using CCCP; in one experiment, some cells could tolerate a very high degree of mitochondrial swelling and undergo MPTP and membrane depolarisation, yet still recover (Minamikawa, Williams, Bowser, & Nagley, 1999).

In fact, the ability of mitochondria to act as temporary calcium stores during signalling in the brain, for instance, following glutamate stimulation of calcium uptake, is critical as poor mitochondrial function can rapidly lead to cell death and loss of function (Nicholls, 2017). It has now also been shown that enhanced mitochondrial respiration can protect against endoplasmic reticulum (ER) stress, a process triggered by calcium, and enhanced mitochondrial biogenesis increased protection further (Knupp et al., 2018). Significantly, plant mitochondria display much of the same morphology and mitochondrial dynamics as animal mitochondria (see figures in [Jaipargas, Barton, Mathur, & Mathur, 2015]), and are also involved in defence against pathogens, as they congregate around infection sites (Fuchs et al., 2016). Hence, re‐establishing a healthy population of mitochondria with a large capacity to buffer calcium would be key in resistance to stress, and the resolution of inflammation. That many plant compounds are anti‐inflammatory, and modulate mitochondrial dynamics, would strongly suggest these observations are linked.

## 5 SUMMARY OF THEORY

### 5.1 Ageing and the loss of complexity

Ageing, it seems, is associated with a gradual loss of the ability to main complexity (Jazwinski & Kim, 2019). Equally, maintenance of efficient electron flow through the mitochondrial ETC to prevent excessive reduction seems to be an evolutionary step to increase lifespan by slowing the ageing process, which, as it fails, is associated with a whole series of age‐related diseases that eventually lead to death (Lane, 2005). In contrast, deliberately redirecting electrons as a stress signal to invoke repair and as an anti‐pathogen strategy in inflammation is also a key function of mitochondria (Banoth & Cassel, 2018; Meyer et al., 2018). Although mounting an immune response is essential for survival, chronic inflammation seems to play a fundamental role in many diseases and is associated with a gradual loss of mitochondrial function (Lane, 2003; Salminen et al., 2008; Salminen, Ojala, Kaarniranta, & Kauppinen, 2012).

In effect, as an organism ages, the ability to maintain a controlled dissipation of energy is slowly lost, which is commensurate with a loss of structure and rising stress. During life, there is a constant process of renewal, in effect, natural selection of the fittest components to maintain this dissipation. However, this process seems to eventually fail leading to ageing and death. Within this paradigm is the concept that order can arise out of chaos if a system is perturbed: this implies that for life to maintain its optimum structure, it has to be constantly exposed to a degree of stress, which could be called hormesis (Nunn, Guy, & Bell, 2017). The bottom line is that although nearly all organisms age, the rate that they age can be varied by the amount and kind of stress they receive; they need enough to ‘remind’ the structure to exist, but too much destroys it.

### 5.2 The role of inflammation resolution and calcium hormesis

One of the observations about many plant extracts is that they are primarily viewed as anti‐inflammatory. However, data suggest at lower concentrations they could actually be inflammatory, and it is only at higher concentrations they become anti‐inflammatory (Schink et al., 2018), suggesting a hormetic process (Calabrese et al., 2019). From the plant's perspective, this supports their role in signalling/adaptation to stress, which probably evolved from their general ability to dissipate solar output and deal with free radicals. Because plants and animals evolved from a common ancestor, and thus contain mitochondria, the basic stress adaptive systems of animals could respond in a similar way.

In general, it could be surmised that the compartmentalisation and timing of calcium signalling could be important; initial ingress of calcium into the cell, or release from the ER, could actually have a mild stimulatory effect—including on the mitochondria. However, this enhancement of mitochondrial function, perhaps coupled with a direct interaction with mitochondria to further enhance mitochondrial uptake of calcium, could act to reduce the proliferative signal. Life relies on the maintenance of a charge across a membrane, which is in effect, a store of energy; most of the time this is driven by extracting energy from electron flow. So, an influx of calcium into the mitochondrion could potentially dissipate this potential, which is presumably why calcium also stimulates mitochondrial function. It could therefore be predicted that compounds that induce calcium flow into a mitochondrion, stimulating its function and thus ROS generation, could be linked to an ability to both alter ROS signalling from complex 1, as well altering ATPase, for instance, to induce uncoupling, would be used to generate a retrograde signal to the nucleus. In fact, mitonuclear signalling can be viewed as being hormetic and bidirectional (Quiros, Mottis, & Auwerx, 2016).

A key outcome of hormesis is therefore to stimulate an increased capacity to flow electrons over time, which would have an anti‐inflammatory effect. This could be achieved by well‐described mitophagy and mitochondrial biogenesis, effectively resulting in newer and fully functional mitochondria. However, there is also evidence that bits of mitochondria can be recycled via generation of vesicles containing damaged components (mitochondrial‐derived vesicles, or MDVs), which may involve VDAC (Roberts, Tang, Fon, & Durcan, 2016). Indeed, it is now becoming clear that MDVs are involved in multiple processes, ranging from inflammation (Puhm et al., 2019) to anti‐bacterial functions (Abuaita, Schultz, & O'Riordan, 2018); the origins of this derive from the observation that prokaryotes also produce vesicles (Toyofuku, Nomura, & Eberl, 2019). The fact that many plant compounds modulate VDAC could thus be part of this adaptive process.

### 5.3 Medicinal plant compounds tune the stress response

It could therefore be argued that these plant compounds can very finely tune the cellular response to stress. Indeed, there does seem to be some evidence that rather than mitochondria being viewed as a giant energy generating unit, they are actually much more likely to consist of multiple linked bioenergetic units based on individual cristae (Wolf et al., 2019), which suggests individual units could fail and be removed. This might also support the underlying hypothesis as to why mitochondria retain some genes to allow for localised redox feedback (Allen, 2015). In relation to this more subtle form of mitochondrial component renewal, we and others have shown that CBD can modulate extracellular vesicle production, both in cancer cells and in bacteria (Kosgodage et al., 2018; Kosgodage, Matewele, et al., 2019; Kosgodage, Uysal‐Onganer, et al., 2019). This may well indicate that vesicle modulation is part of how CBD may be working.

In terms of the stress response, one of the most important signalling hubs in all eukaryotes is the target of rapamycin (TOR) complex, which seems to be critical in interpreting environmental and internal signals to control the switch between stress resistance and growth, and thus, determining lifespan. New data is now showing that it can be associated with MAMs and VDAC1 (Betz et al., 2013; Ramanathan & Schreiber, 2009), and is now thought to play an integrated role with mitochondria in determining longevity (Wei, Zhang, Cai, & Xu, 2015). As it fulfils many of the same functions in plants (Rodriguez, Parola, Andreola, Pereyra, & Martinez‐Noel, 2019), this might suggest that these plant medicinal compounds can also modulate the TOR complex in animals. It is therefore of interest that phenolic terpenoids, such as thymol and carvacol, which can inhibit fungal growth, seem to mimic calcium stress by inducing calcium ingress and inhibiting mTOR (Rao, Zhang, Muend, & Rao, 2010). Thymol can also alter mitochondrial function and enhance ROS (Deb, Parimala, Saravana Devi, & Chakraborty, 2011). It is thus of relevance that TORC2, a complex of TOR, seems to inhibit calcineurin by suppressing mitochondrial ROS and the activation of the calcium channel regulatory protein, Mid 1 (Vlahakis, Lopez Muniozguren, & Powers, 2017).

### 5.4 Prefactor chromophores and modern sunscreens

**Figure 3:**
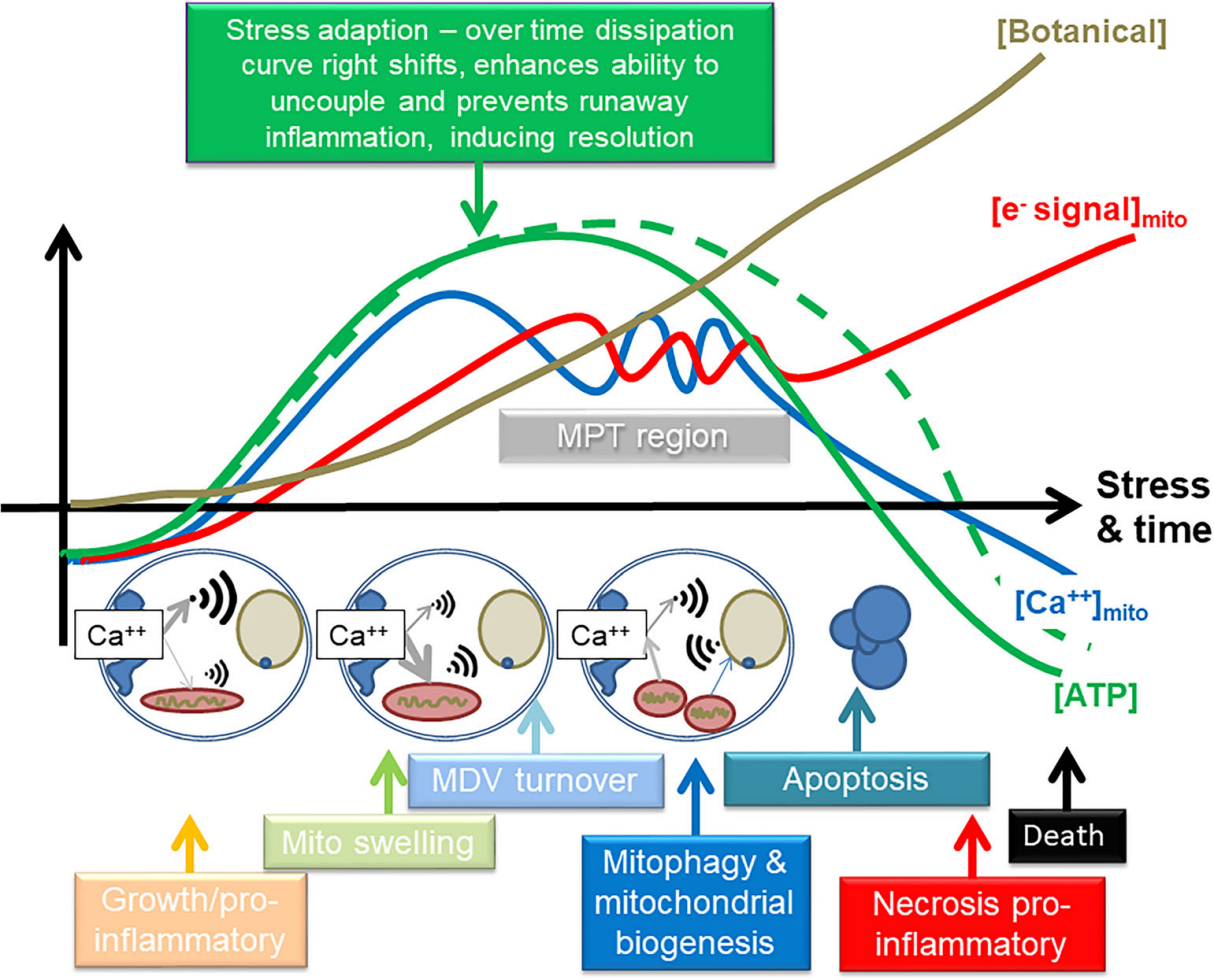
Biphasic effects of plant compounds on mitochondrial function. This diagram shows a calcium‐centric viewpoint. It is possible that plant compounds were originally sunscreen/electron dissipaters, but through evolution, became part of a broader stress response system that both directly and indirectly modulated mitochondrial function. Flow of calcium into a mitochondrion would stimulate function, but would also eventually inhibit function, and would provide a powerful internal mitochondrial signal to renew damaged complexes, as well as a retrograde signal to the nucleus and other parts of the cell (e.g.,mitochondrial biogenesis and autophagy, and upregulation of anti‐oxidant pathways). This could be achieved in a number of ways, both via modulation of calcium channels in the plasma and ER membranes, as well on the mitochondrion itself – many of these compounds are both lipophilic and could take part in redox. They could also directly influence the ETC, as well as potentially the ATPase, which could fine tune the signalling process – so modulating ROS and uncoupling. The dose would be critical, and it is likely that the system evolved where low doses would start by “priming” the system, possibly by activating redox sensitive growth pathways that inhibit cell death, but as dose increased, they would inhibit this process to ensure enhanced turnover and over‐compensation of capacity to ensure resolution. At still higher concentrations, they would be capable of inducing cell death, both of self‐cells, but also of pathogens. It is also possible, depending on where they end up, and depending on the type of molecule and wavelength, absorb light and provide some protection against UV. Key: MPT = mitochondrial permeability transition; MDV = mitochondrially derived vesicles; blue line = mitochondrial calcium concentration; red line = electron leak leading to ROS; green line = dissipation & ATP; brown line = possible plant compound concentration

To conclude, it is possible that the natural selection of chromophoric molecules that originally could dissipate the energy in light, both as photons or electrons, has led to them becoming key moieties in a more generalised stress‐signalling hormetic system, that certainly in eukaryotes, is focussed around mitochondrial function. This system may well parallel a much more ancient set of circumstances that led to life itself. It could be said that medicinal plant compounds engender a negative feedback shift leading to structural stabilisation through dissipation. Figure 3 summarises this idea, and displays it against the well‐described relationship between calcium and mitochondrial function.

## CONFLICT OF INTEREST

The authors declare no conflicts of interest.

## AUTHOR CONTRIBUTIONS

The concept was discussed by A.V.N., G.W.G., and J.D.B. A.V.N. developed the theory and wrote and edited the manuscript. G.W.G., S.W.B., and J.D.B. provided feedback on each draft of the manuscript and approved the final version.
